# On the Mammalian Nervous System

**Published:** 1892-09

**Authors:** 


*On the Mammalian Nervous System.* By FRANCIS GOTCH, Hon. M.A. Oxon., and VICTOR HORSLEY, F.R.S. London: KEGAN PAUL, TRENCH, TRÜBNER and Co. 1891.

Without giving a lengthy abstract, which the limits of our space unfortunately preclude us from doing, we cannot give a full idea of the value of this research. We must therefore content ourselves with saying that no more important contribution to the physiology of the brain and spinal cord has for a long time appeared. It embodies the results of a long-continued and laborious investigation into the action of nervous centres and the mode of conduction of impulses along the spinal cord, for the study of which a most ingenious and delicate method of research was conceived and carried out by the authors. We may note the confirmation that their experiments bring to the well-known views of Professor Bastian as to the constitution of the nerve centres, and, with regard to the spinal cord, the important fact that afferent impulses are to the extent of 80 per cent. conducted upwards along the same side of the cord as that on which they reach it. For further details we must refer our readers to the book itself, merely adding that it is one which those who are interested in the study of neurology must read, mark, and digest; and at the same time we must congratulate the authors on the very important addition that their labours have made to our previous knowledge of the subject.

This work, which we have gladly received as a separate volume, also forms part of Vol. 182 of the *Philosophical Transactions of the Royal Society of London.*

